# The Correlation Between Triglyceride‐Glucose Index (TyG Index) With the Severity of Acute New‐Onset Heart Failure (NOHF) Complications in Acute Myocardial Infarction Based on Killip Classification

**DOI:** 10.1155/crp/6997679

**Published:** 2026-04-06

**Authors:** Levina Felicia, Nida Suraya, Ratu Purwanti

**Affiliations:** ^1^ Department of Clinical Pathology, Faculty of Medicine, Universitas Padjadjaran, Bandung, Indonesia, unpad.ac.id

**Keywords:** acute myocardial infarction, acute new-onset heart failure, Killip classification, TyG index

## Abstract

**Background:**

Cardiovascular disease is the leading cause of morbidity and mortality in Indonesia, in which acute myocardial infarction (AMI) has an estimated mortality rate of 9.2%. Heart failure is the most common complication of AMI and increases the risk of death by 3‐4 times. The Killip classification assesses the severity and prognosis of acute new‐onset heart failure (NOHF) in AMI patients: Class I (6% mortality), Class II (17%), Class III (38%), and Class IV (81%). The triglyceride‐glucose (TyG) index, a reliable marker for insulin resistance, is linked to cardiovascular disease pathogenesis. Several studies showed that the TyG index can predict the development of cardiovascular events.

**Aim:**

This study aims to determine the correlation between TyG index levels and the severity of acute NOHF complications in patients with acute myocardial infarction based on the Killip classification.

**Methods:**

This cross‐sectional analytical observational study involved adult patients with AMI diagnoses in the ER of Dr. Hasan Sadikin Hospital, Bandung, in January–December 2023, who had fasting blood glucose and triglyceride examinations within 72 h of admission and had been assessed with Killip classification. Data analysis was performed using the Spearman test using SPSS software version 25.0.

**Results:**

This study included 100 AMI patients who met the inclusion and exclusion criteria. Significant relationships were found between comorbidities (diabetes mellitus, hypertension, and smoking) and patient outcomes against Killip classification. Killip Class I had the most improved outcomes (54 patients). Killip Class IV (10 patients) had the most deaths and highest triglycerides and glucose levels. The TyG index showed a strong positive correlation with acute NOHF severity in patients with AMI based on the Killip classification (*r* = 0.746, *p* < 0.001).

**Conclusion:**

The TyG index has a strong positive correlation with the severity of acute NOHF in patients with acute myocardial infarction based on the Killip classification.

## 1. Introduction

The leading cause of morbidity and mortality in Indonesia is cardiovascular disease. Indonesia is a country with the highest burden of cardiovascular disease in Southeast Asia. Acute coronary syndrome accounts for most of the cardiovascular diseases in Indonesia, and its mortality rate was 9.2% in 2017 [1, 2]. The Ministry of Health of the Republic of Indonesia reported that cardiovascular disease ranks first place among the eight highest catastrophic diseases in Indonesia and is the most funded disease by the Social Health Insurance Administration Body [3].

Acute coronary syndrome includes conditions ranging from unstable angina pectoris (UAP) to acute myocardial infarction, either with ST‐segment elevation (STEMI) or without ST‐segment elevation (NSTEMI) [4, 5]. Acute myocardial infarction is the leading cause of cardiovascular disease morbidity and mortality globally [6]. The most common complication in patients with acute myocardial infarction is acute heart failure (HF), with an incidence of approximately 50% among hospitalized patients [5, 7].

Acute new‐onset HF (NOHF) is an acute HF that occurs for the first time without any history of heart disease [8]. Acute NOHF that occurs due to acute myocardial infarction has a 3 to 4‐fold higher risk of mortality. The severity and prognosis of acute NOHF in acute myocardial infarction patients are commonly evaluated by clinicians using the Killip classification. In Class I Grade classification, patients do not have HF that is characterized by rhonchi or additional heart sounds, and its mortality rate is 6%. Grade II Killip classification is associated with mild to moderate HF, and its mortality rate is 17%. Acute lung edema is found in the Class III Killip classification and its mortality rate is 38%. Grade IV Killip classification, is the most severe, in which cardiogenic shock is found, and its mortality rate reaches 81% [5]. Therefore, the prevention and screening of myocardial infarction patients are crucial because late or missed diagnosis of acute HF have adverse effects on the patients’ prognosis and increase the therapeutic burden [9–12].

The triglyceride‐glucose index (TyG index) is the latest replacement index to detect insulin resistance, and it is widely known that the TyG index plays an important role in cardiovascular disease pathogenesis [13–15]. The gold standard diagnostic method for insulin resistance is hyperinsulinemia‐euglycemia clamp; however, this technique is complex, time‐consuming, expensive, and difficult to be applied clinically. A fast, rapid, and applicative marker is required for insulin resistance assessment and its predisposition to cardiovascular diseases. The TyG index has higher sensitivity and specificity (sensitivity of 96.5% and specificity of 85%) than hyperinsulinemia‐euglycemia clamp [16]. Several studies have demonstrated that the TyG index is associated with coronary artery calcification, atherosclerosis, and high risk of ACS [17–19]. Sánchez‐Iñigo et al. concluded that the TyG index can predict cardiovascular disease development [14]. However, when the TyG index is associated with HF complications, the correlation between the TyG index with the severity of HF complication in acute myocardial infarction patients based on the Killip classification is not widely known. Therefore, researchers were interested to learn further about the correlation between the TyG index with the severity of acute NOHF complications based on the Killip classification.

## 2. Methods

The study was conducted in the Central Laboratory Installation of Dr. Hasan Sadikin Central General Hospital, Bandung, Indonesia, from April 2024 to July 2024. The participants of this study were adult patients with an acute myocardial infarction (International Classification of Diseases [ICD] I21) diagnosis that was established through clinical examinations, electrocardiography confirmation, and cardiac markers and who came to the emergency department of Dr. Hasan Sadikin Central General Hospital from January 2023 to December 2023. The sampling method in this study was total sampling, in which all acute myocardial infarctions that fulfilled the inclusion and exclusion criteria were included in the study. The inclusion criteria were adult patients (aged > 18 years) diagnosed with STEMI or NSTEMI based on EKG and Troponin I in the Emergency Department and underwent random blood glucose and fasting blood triglyceride levels within 72 h after admission. The Killip classification is applied when patients come to the Emergency Department. The exclusion criteria are patients with a history of heart diseases such as prior acute myocardial infarction, congestive HF, heart valve disorders, congenital heart disease, history of chronic kidney disease, patients with malignant disease, patients with autoimmune disease, and patients with incomplete and inaccessible data during the research period.

This was an analytic observational study with a cross‐sectional design. The data were retrospectively collected using the Hospital Information System and Laboratory Information System of Dr. Hasan Sadikin Hospital from January 2023 to December 2023. The TyG index levels would be classified based on the Killip classification. The TyG index was calculated using the following formula: In ((fasting blood triglyceride (mg/dL) × fasting blood glucose (mg/dL))/2. Clinicians will determine the grade of Killip classification based on their clinical conditions. Patients were assigned to Grade I Killip classification if there were no clinical signs of HF; Grade II Killip classification if there was HF characterized by wet rhonchi in the half lung field, additional heart sounds, and increased jugular vein pressure; Grade III classification if there was acute lung edema; and Grade IV Killip classification if there was cardiogenic shock, characterized by systolic blood pressure < 90 mmHg and tissue hypoperfusion signs (e.g., urine output < 30 mL/h, cool extremities, altered mental status, and/or serum lactate > 2.0 mmol/L).

Numerical data were presented as mean ± standard deviation for normally distributed variables and as median (minimum–maximum) for non‐normally distributed variables. The categorical data were presented in percentage (%) for each category. All data were presented in tables. The data normality was determined using the Kolmogorov–Smirnov test because the sample size was more than 50. Bivariate analysis was conducted to assess the correlation between the TyG index and the Killip classification. Correlation analysis was performed using Spearman’s rank correlation test. The *p* value of < 0.05 was considered statistically significant, the correlation strength was determined statistically from the correlation coefficient (*r*). The data were analyzed using Statistical Product and Service Solution (SPSS) for Windows version 25.0.

This study has been approved by the Research Ethical Committee of the Faculty of Medicine, Padjadjaran University/Hasan Sadikin Central General Hospital, Bandung (DP.04.03/XIV.6.5.198/2024).

## 3. Results

The total number of acute myocardial infarction patients in the Emergency Department of Hasan Sadikin Bandung General Hospital was 308 people in 2023. Of the 308 patients, 208 were excluded; therefore, 100 patients who fulfilled the inclusion criteria were included in the study. The baseline characteristic of included acute myocardial patients based on the Killip classification is presented in Table 1.

**TABLE 1:** The baseline characteristics of participants based on Killip classification.

Variable	Killip classification				*p*
	Killip I *n* = 54	Killip II *n* = 14	Killip III *n* = 18	Killip IV *n* = 14	
Age (year)					
Mean ± SD	55.5 ± 9.8	65.5 ± 11	58.3 ± 10.8	58.9 ± 8.8	0.171
Gender, *n* (%)					
Men	48 (88.9)	11 (78.6)	14 (77.8)	11 (78.6)	0.173
Women	6 (11.1)	3 (21.4)	4 (22.2)	3 (21.4)	
Diagnosis, *n* (%)					
STEMI	45 (83.3)	13 (92.9)	13 (72.2)	11 (78.6)	0.503
NSTEMI	9 (16.7)	1 (7.1)	5 (27.8)	3 (21.4)	
Comorbid, *n* (%)					
Diabetes mellitus	6 (11.1)	2 (14.3)	6 (33.3)	5 (35.7)	0.012^∗^
Hypertension	19 (33.3)	8 (57.1)	11 (61.1)	7 (50)	0.038^∗^
Smoking history	44 (81.5)	6 (42.9)	8 (44.4)	10 (71.4)	0.015^∗^
Outcome, *n* (%)					
Improved	54 (100)	13 (92.9)	14 (77.8)	4 (28.6)	
Deceased > 48 h	0 (0)	1 (7.1)	2 (11.1)	9 (64.3)	< 0.001^∗^
Deceased < 48 h	0 (0)	0 (0)	2 (11.1)	1 (7.1)	
Laboratory					
Fasting blood triglyceride (mg/dL)	89 (35–275)	124 (80–207)	150.5 (86–400)	174 (104–691)	< 0.001^∗^
Fasting blood glucose (mg/dL)	100 (61–181)	113 (84–196)	138 (94–168)	179 (133–510)	< 0.001^∗^
TyG index	4.6 (4–5.4)	4.8 (4.6–5)	5 (4.7–5.4)	5,2 (4.8–5.9)	< 0.001^∗^
Routine Medications	Yes			No	
Statin	87 (87%)			13 (13%)	
Antihypertension	92 (92%)			8 (8%)	
Antidiabetic	95 (95%)			5 (5%)	

*Note:* The normally distributed ones were presented in mean ± SD while the not normally distributed ones were presented in median (minimum–maximum).

^∗^ *p* < 0.05 was considered statistically significant.

Table 1 demonstrates that most of the acute myocardial infarction participants in all grades of the Killip classification were male. The mean age of myocardial infarction patients across all Killip classes was > 55 years. Most acute myocardial infarctions in Killip Grades I, II, III, and IV were diagnosed as STEMI. Overall, most participants across Killip grades and TyG index had percutaneous intervention (PCI) and had routine medications including statins (87%), antihypertension (92%), and antidiabetic (95%).

The most common comorbid diseases in this study were hypertension and smoking. There were significant correlations between diabetes mellitus, hypertension, and smoking status with Killip classification (all *p* < 0.05). Furthermore, there was a significant correlation between patient outcomes with Killip classification in which the highest number of patients with improvement and deceased were found in Grade I Killip (54 patients) and Grade IV (10 patients), respectively (*p* < 0.001).

Grade IV Killip acute myocardial infarction patients had the highest median fasting blood triglyceride level (174 mg/dL, *p* < 0.001), fasting blood glucose level (179 mg/dL, *p* < 0.001), and TyG index (5.2, *p* < 0.001).

**FIGURE 1:**
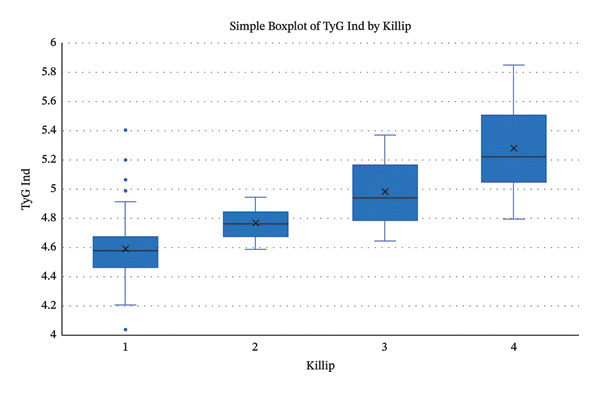
The boxplot of TyG index to Killip classification.

The proportion of the TyG index based on the Killip classification is presented in Figure 1. The correlation between the TyG index with the severity of acute NOHF complications in acute myocardial infarction patients was analyzed in this study, and the analysis results were presented in Table 2.

**TABLE 2:** The correlation of laboratory examination and postmyocardial infarct (MI) ejection fraction with the severity of acute new‐onset heart failure (NOHF) complications in acute myocardial infarction on patients based on Killip classification.

Variables	Killip classification	
	*r*	*p*
Fasting blood triglyceride	0.591	< 0.001^∗^
Fasting blood glucose	0.622	< 0.001^∗^
TyG index	0.746	< 0.001^∗^
Post‐MI ejection fraction	−0.436	< 0.001^∗^

^∗^ *p* < 0.05 was considered statistically significant.

The analysis was conducted using the Spearman correlation test. There was a significant positive correlation between fasting blood triglyceride levels, random blood glucose, and TyG index with the severity of acute NOHF complications in acute myocardial infarction patients based on Killip classification. The TyG index had the strongest correlation among other variables (*r* = 0.746, *p* < 0.001). Post‐MI ejection fraction (EF) had a negative correlation with Killip classification (*r* = −0.436, *p* < 0.001).

**FIGURE 2:**
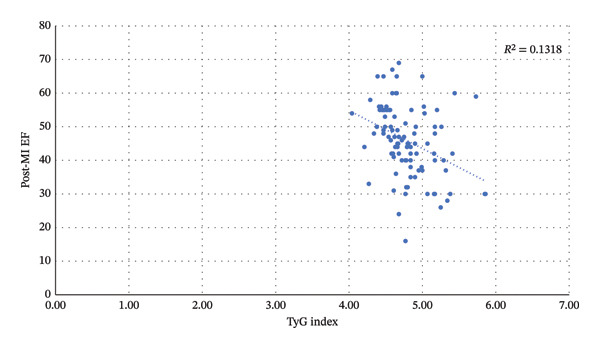
The scatter‐plot diagram of TyG index to post‐MI EF.

Further analysis was conducted to evaluate the relationship between the TyG index and post‐MI EF. This analysis was performed using Spearman correlation and demonstrated a significant association (*p* < 0.001). The *r*‐value indicates a negative correlation (*r* = −0.363), suggesting that a higher TyG index is associated with a decline in heart function. Figure 2 shows the scatter plot illustrating this correlation. Although the correlation between the two variables is weak, it is important to note that triglyceride control is pivotal for maintaining good heart function.

An analysis of the association between the post‐MI EF and Killip classification was conducted using the Kruskal–Wallis test, as the data were not normally distributed. The post‐MI EF was significantly associated with Killip classification (*p* = 0.011). Post hoc analysis using Dunn’s test revealed significant differences between Killip classes I and III, as well as between Classes I and IV. The results of the analysis are presented in Tables 3 and 4.

**TABLE 3:** Kruskal–Wallis test between post‐MI EF and Killip classification.

Killip class	Post‐MI ejection fraction		*p* value
	Mean ± SD	Median (IQR)	
1	48.94 ± 8.13	49.00 (12.00)	0.011
2	44.79 ± 11.78	44.00 (16.00)	
3	41.67 ± 11.43	40.00 (14.00)	
4	40.86 ± 13.09	37.50 (22.00)	

**TABLE 4:** Dunn’s test between post‐MI EF and Killip classification.

Killip class (a)	Killip class (b)	Mean difference (a‐b)	*p* value
1	2	4.16	0.120
	3	7.28	0.102
	4	8.09	0.072

2	3	3.12	0.154
	4	3.93	0.131

3	4	0.81	0.928

**FIGURE 3:**
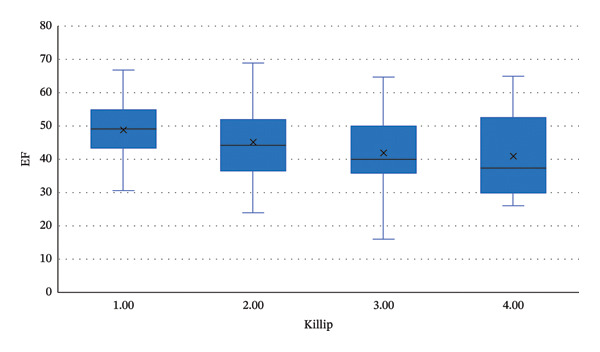
Simple boxplot of post‐MI EF by Killip class.

Based on the results presented above, Killip Class I had the highest EF compared to the other classes. A linear pattern was observed, with EF decreasing as the Killip class increased. The lowest EF was found in Killip Class IV (40.86 ± 13.09). The difference in EF was not significant when comparing Killip Class I to Killip Class III, with a mean difference of 7.28 (*p* = 0.102). An even greater difference was observed between Killip Class I and Killip Class IV, calculated at 8.09 (*p* = 0.072). The simple boxplot diagram is shown below in Figure 3.

## 4. Discussion

Most participants in this study were male. Previous studies stated that gender has a significant association with in‐hospital mortality of acute myocardial infarction patients [7]. Reproductive‐age women often have a lower risk of myocardial infarction compared to men. Estrogen has protective effects on heart disease. However, the risk of acute myocardial infarction significantly increased in women after menopause, parallel to the men’s risk. The finding in this study is in accordance with this theory; all women with acute myocardial infarction in this study were menopausal women [7, 20]. The most common acute myocardial infarction is STEMI. This finding is similar to a study in France that 61.5% of acute coronary syndrome patients were STEMI patients [21].

Table 1 reveals statistically significant correlations between diabetes mellitus, hypertension, and smoking with the grade of Killip classification. This finding is similar to the study by Itzahki et al. [22], patients with Grades II–IV Killip tended to have comorbid such as smoking, diabetes mellitus, and hypertension [23]. Hashmi et al. [7] also revealed that diabetes mellitus and smoking were associated with Killip classification. El‐Menyar et al. [23] also have similar findings. Diabetes mellitus, hypertension, and smoking increase the chance of increased risk and higher grade of Killip classification [23].

This study also found that there was a statistically significant correlation between clinical outcomes with Killip classification. The severity of acute NOHF complications in acute myocardial infarction patients is associated with the Killip classification. Most of the patients who died > 48 h were Grade IV Killip patients, and some patients who died < 48 h were Grades III and IV Killip patients. The Killip classification is a clinical assessment in patients with acute myocardial infarction, and it can assess the severity of HF after myocardial infarction. The Killip classification is proven as a good, reliable, and valid tool for early risk stratification in acute myocardial infarction patients. It also has a prognostic role in patients’ mortality [7, 24–26]. The mortality rate increased progressively with higher Killip classes [7]. Patients with Grade IV Killip have a 16‐fold higher risk of in‐hospital mortality [27].

The correlation test revealed that triglyceride levels, fasting blood glucose, and TyG index had a positive correlation with the Killip classification. The results of the further analysis revealed that the TyG index had a positive and significant correlation with the severity of acute NOHF complications in acute myocardial infarction based on the Killip classification (*r* = 0.746; *p* < 0.001). The TyG index had a better correlation compared to fasting blood glucose (*r* = 0.622, *p* < 0.001), and fasting blood triglyceride (*r* = 0.591, *p* < 0.001). This finding is similar to several studies that also found that an increased TyG index was correlated with the incidence, severity, and mortality of patients with various cardiovascular diseases including acute myocardial infarction. A study by Liang et al. [28] found that patients with higher TyG indices have a higher risk of coronary heart disease, greater coronary artery lesions, and poor prognosis compared to patients with lower TyG indices. Luo et al. [29] stated that the TyG index can predict the severity of coronary heart disease and cardiovascular outcomes independently in STEMI patients [29]. A study by Zhang et al. found that a high TyG index was associated with the incidence of acute myocardial infarction and larger myocardial infarction size; therefore, high TyG index is associated with the severity of coronary heart disease [30].

The TyG index is an insulin resistance marker that is closely related to the risks of cardiovascular diseases and contributes to cardiovascular disease development through two distinct pathways, atheroma plaque formation and diastolic abnormality and ventricular hypertrophy [29]. Insulin resistance will lead to glucose metabolism disorder that can trigger hyperglycemia. Glucotoxicity can lead to inflammation and oxidative stress. Furthermore, the lipid metabolism disorder that is characterized by increased triglyceride and low‐density lipoprotein (LDL) along with decreased high‐density lipoprotein (HDL) can trigger lipotoxicity conditions. This condition will initiate atherosclerosis which plays an important role in acute myocardial infarction. Insulin resistance also can lead to increased sympathetic nervous system activity and heart burden along with hyperinsulinemia. Renin‐angiotensin‐aldosterone system (RAAS) activation by insulin resistance will interfere the heart function [31]. These processes lead to cardiomyocyte damage and death, heart hypertrophy, and heart fibrosis which were important pathologic factors in HF [16, 32–35].

Hao et al. also found that patients with a high TyG index had a higher incidence of HF. Acute myocardial infarction patients with a TyG index > 9.23 had a ninefold higher risk of HF. Other than HF, the TyG index is also related to coronary microvascular dysfunction in AMI patients, one of the possible reasons for poor prognosis in AMI patients. A high TyG index reflects a higher body mass index (BMI), which is associated with a relatively higher cardiac load, and patients with higher BMIs were more prone to having decompensated HF after AMI‐causing cardiac events [36]. Therefore, the TyG index can be a simple prognostic marker for HF complications in acute myocardial infarction, considering that early risk stratification is crucial to prevent deterioration and optimize patient management.

The TyG index also had a predictive value for STEMI patients who underwent PCI. Ebaid et al. found that a higher TyG index was associated with poor in‐hospital outcomes in STEMI patients who underwent PCI, including in‐hospital mortality [37]. A meta‐analysis by Liang et al. found that the TyG index positively correlated with the risk of coronary artery disease and stenotic coronary arteries [28]. Liu et al. also demonstrated that the TyG index positively correlated with myocardial infarction, cardiovascular disease, and coronary artery disease incidence in general populations [38]. Those studies highlighted that the TyG index not only has prognostic value for HF complications among STEMI patients but also is a predictor of CVD risks among the general population, although further studies are necessary.

Clinicians may apply the TyG index as a noninvasive method to assess the metabolic state of patients, particularly TyG‐BMI, which has implications for lifestyle modifications such as diet and exercise to improve insulin sensitivity, which are also associated with CVD risks [39]. We also found that post‐MI EF was negatively correlated with Killip classification. This finding aligns with Liu et al., who found that higher Killip grades had lower EF, which means that higher Killip grades were correlated with poor cardiac function [40]. A higher TyG index reflects higher BMI and insulin resistance that increase oxidative stress and inflammation, leading to cardiomyocyte apoptosis and endothelial dysfunction, thereby reducing the deformation capacity of myocardium, damaging myocardium, and decreasing myocardial function at the end [41]. The limitation of this study is an uneven distribution of each grade of Killip classification. A larger sample size in every grade of Killip classification will result in statistical results that are closer to the real statistic value.

## 5. Conclusion

The TyG index has a strong and significant positive correlation with the severity grade of NOHF complications in patients with acute myocardial infarction based on Killip classification. Further studies should have samples that are equally distributed in each grade of Killip classification for further assessment of TyG index potency as an acute myocardial infarction prognosis and further learning about the correlation of TyG index with HF complications in acute myocardial infarction.

## Funding

The authors did not receive funding from any person, institution, or organization.

## Conflicts of Interest

The authors declare no conflicts of interest.

## Data Availability

The data that support the findings of this study are available on request from the corresponding author. The data are not publicly available due to privacy or ethical restrictions.
